# Variants of Chaotic Grey Wolf Heuristic for Robust Identification of Control Autoregressive Model

**DOI:** 10.3390/biomimetics8020141

**Published:** 2023-03-30

**Authors:** Khizer Mehmood, Naveed Ishtiaq Chaudhary, Zeshan Aslam Khan, Khalid Mehmood Cheema, Muhammad Asif Zahoor Raja

**Affiliations:** 1 Department of Electrical and Computer Engineering, International Islamic University, Islamabad 44000, Pakistan; 2 Future Technology Research Center, National Yunlin University of Science and Technology, 123 University Road, Section 3, Douliou 64002, Taiwan; 3 Department of Electronic Engineering, Fatima Jinnah Women University, Rawalpindi 46000, Pakistan

**Keywords:** chaos, parameter estimation, ARX, grey wolf optimizer

## Abstract

In this article, a chaotic computing paradigm is investigated for the parameter estimation of the autoregressive exogenous (ARX) model by exploiting the optimization knacks of an improved chaotic grey wolf optimizer (ICGWO). The identification problem is formulated by defining a mean square error-based fitness function between true and estimated responses of the ARX system. The decision parameters of the ARX model are calculated by ICGWO for various populations, generations, and noise levels. The comparative performance analyses with standard counterparts indicate the worth of the ICGWO for ARX model identification, while the statistical analyses endorse the efficacy of the proposed chaotic scheme in terms of accuracy, robustness, and reliability.

## 1. Introduction

Parameter estimation plays an important role in system identification, which is the frontier of research in signal processing [1]. It is widely applied in various applications such as Hammerstein autoregressive system [2], water turbine [3], electrical machines [4], fuel cells [5], recurrent neural networks [6], health [7], Hammerstein–Wiener system [8], computer-aided design [9], renewable energy resources [10], honey production [11], Magnetorheological dampers [12], and smart grids [13]. Various techniques were proposed in the literature related to parameter estimation such as metaheuristics [14], fractional algorithms [15], least mean square [16], fuzzy logic [17], analytical methods [18], and machine learning [19].

**Figure 1:**
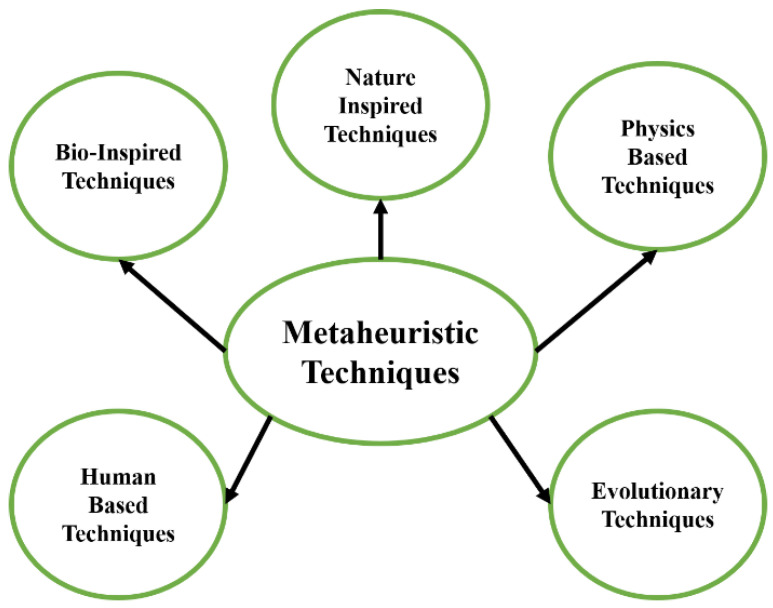
Classification of metaheuristic techniques.

Among these techniques, metaheuristics have gained significant attraction in recent decades for system identification. As presented in Figure 1, metaheuristic techniques are classified into five domains. The first domain is bio-inspired techniques, and various techniques are proposed in this domain. The particle swarm optimization (PSO) [20] is inspired by the movement and intelligence of swarms. The artificial rabbits optimization [21] is inspired by the survival strategies of rabbits, which include detour foraging and random hiding. The grey wolf optimization (GWO) [22] mimics the behavior of grey wolves for hunting and leadership hierarchy. Manta ray foraging optimization [23] mimics the three unique strategies of manta rays, which includes chain, cyclone, and somersault for solving optimization problems. Artificial hummingbirds [24] use flight skills and foraging strategies of hummingbirds.

The second domain is human-based techniques which were used for optimization. In teaching–learning-based optimization [25], inspired by a classroom environment in which optimal solution is calculated by knowledge sharing between teacher and students. In city councils evolution [26], the councils evolved from smallest to largest neighborhoods. Based on the performance of council members, they became members of the larger councils. The mountaineering team-based optimization [27] is inspired by the leader’s experience of guided and regular movement by climbers to reach the mountain top. In political optimizer [28], the optimization solution is obtained by considering each party member as a solution, and their election behavior is used for evaluation. In the parliamentary optimization algorithm [29], intra- and inter-group competitions are conducted for taking control of parliament.

The third domain is evolutionary techniques applied to optimization problems. In differential evolution [30], an optimization solution is obtained by using mutation, crossover, and selection operators. In egret swarm optimization [31], sit-and-wait strategy, aggressive strategy, and discriminant conditions were used for finding the optimal solution. Genetic algorithm [32], uses the concept of genetics and natural selection for solving optimization problems. The evolutionary mating algorithm [33] adopts Hardy–Weinberg equilibrium and crossover index in finding solutions to optimization problems.

The fourth domain includes physics-based techniques applied for optimization. In big bang big crunch [34], two phases, namely, big bang and big crunch, were used for randomness and ordered particle distribution in solving optimization problems. In the circle search algorithm [35], features of a circle such as a diameter, radius, perimeter, tangent lines, and angle were used for obtaining optimization solutions. Newton’s metaheuristic algorithm [36] uses Newton’s gradient-based method for population update and incorporates a term containing the best solution in its update rule. Transit search [37] uses the exoplanet exploration method for finding the best optimal solution.

The fifth domain is nature-inspired techniques used in optimization. In the water cycle algorithm [38], the behavior of water flow in rivers, streams, and the sea is formulated for solving optimization problems. Farmland fertility [39] divides farmland into different parts for increasing optimal efficiency in internal and external memory. Sunflower optimizer [40] mimics the movement of sunflowers towards the sun by aggregating the distance between the sun for finding the best solution. In wind-driven optimization [41], velocity and position are updated based on atmospheric motion.

Chaos theory relates the chaotic dynamics of systems with initial conditions and unstable periodic motions [42]. It is applied in various applications such as biometric security [43], embedded systems [44], communications [45], lasers [46], pumped storage units [47], encryption systems [48], the Internet of Things [49], image processing [50], and image encryption [51].

Combining chaos in metaheuristics increases the exploration and exploitation of optimization techniques. Various chaotic metaheuristics were presented in the literature. In [52], a chaotic biogeography-based optimizer is proposed in which chaotic maps were incorporated in the migration, selection, and mutation operations of the optimizer. In [53], an improved version of manta ray foraging called the elite chaotic manta ray algorithm is proposed in which chaotic maps and opposition-based learning are implemented so that it does not fall in local minima. In [54], a chaotic version of the bonobo optimizer is proposed and applied for optimal power flow analysis in renewable energy sources. In [55], a chaotic variant of the salp search algorithm is used for the solution of the economic dispatch problem for different combinations of renewable energy resources. In [56], a chaotic variant of fruit fly optimization is proposed which incorporates fourteen chaotic maps and is tested on ten benchmark problems. In [57], an enhanced version of kill herd optimization is proposed by incorporating sine, circle, and tent chaotic maps. In [58], a chaotic version of invasive weed optimization is proposed for solving optimization problems. In [59], a chaotic quasi-oppositional arithmetic optimization algorithm is proposed for the thermo-economic design of tube and shell. In [60], a chaotic billiards optimization is proposed for optimum parameter estimation of solar hydrogen variable speed induction motor.

Grey wolf optimizer (GWO) has gained significant attention in recent years due to its flexibility, scalability, and few parameters [61]. It is applied in various applications such as gait analysis [62], structural strain reconstruction [63], engines [64], renewable energy systems [65], robotics [66], deep learning [67], wireless sensor networks [68], smart grid [69], medical [70], and energy management [71]. Even though GWO has been utilized in different applications, due to the complexity of real-world optimization problems, various improvements have been made in GWO in terms of updating mechanisms, hybridization, encoding schemes, multi-objective, and new operators.

In [72], a modified GWO for a wireless sensor network is presented. In this work, the weights are dynamically updated based on the distance between the wolves, their prey, and coefficient vectors for improving the optimization ability of GWO. In [73], a chaotic GWO is proposed for solving optimization problems. In this work, chaotic maps were incorporated into GWO for accelerating its convergence. Afterward, it is applied to thirteen constrained benchmark problems and five engineering-constrained problems. In [74], an improved GWO is proposed by incorporating variable weights along with a new governing equation for controlling parameters. In [75], a hybrid version of GWO is proposed. In this work a hybrid sparrow search algorithm GWO is proposed and applied for gain optimization of the proportional–integral–derivative controller. In [76], a hybrid algorithm called GWOPSO is proposed and applied for optimal parameter estimation of the proportional–integral–derivative used for the controlled pump-motor servo system. In [77], an improved chaotic GWO (ICGWO) is proposed by incorporating an adaptive convergence factor and chaotic map in GWO which is further applied in the extraction of supercritical carbon dioxide from a multi-herbal formula.

The autoregressive exogenous model (ARX) is used in different engineering problems such as time series data prediction [78], pneumatic positioning systems [79], wheeled robots [80], multiple-input–multiple-output (MIMO) systems [81], and human driving behavior modeling [82]. Various identification techniques were proposed for the parameter estimation of ARX. In [83], a modified momentum gradient descent algorithm is proposed which uses two gradient directions and sizes in each iteration for ARX identification. In [84], a recursive least squares, decomposition least squares, and interval-varying least squares were used for ARX identification. In [85], dwarf mongoose optimization is used for system identification of the ARX model. In [86], multi-innovation fractional least mean squares were used in estimation. In [87], an Aquila optimizer is used in parameter estimation of the ARX model, In [88], Kalman filter-based multi-step length gradient iterative algorithm with missing outputs is used for parameter estimation of the ARX models. In [89], a Renyi square error entropy and fourth-order statistic of the error–kurtosis–into the variable step size input for used ARX model identification.

The current study is a novel investigation exploring the potential of chaotic maps through an ICGWO for effective parameter estimation of ARX structure. The innovative contributions of the proposed study are as follows:

- The parameter estimation problem of a system represented by the ARX model is investigated through optimization knacks of an improved chaotic grey wolf optimizer (ICGWO).
- The performance of the proposed ICGWO scheme is examined in detail through comparison with the conventional counterparts for various generations, populations, and noise levels.
- The statistical analysis through multiple independent trials confirms the accurate and robust performance of the ICGWO over the GWO, CGWO, and AGWO.
- The accurate estimation for a practical example of a temperature process system further validates the convergent performance of the ICGWO.

The remainder of the article is structured as follows: ARX mathematical structure is presented in Section 2. In Section 3, the ICGWO-based proposed scheme is provided. Section 4 presents the performance comparison of ICGWO, GWO, AGWO, and CGWO. The article is concluded in Section 5.

## 2. ARX Mathematical Model

**Figure 2:**
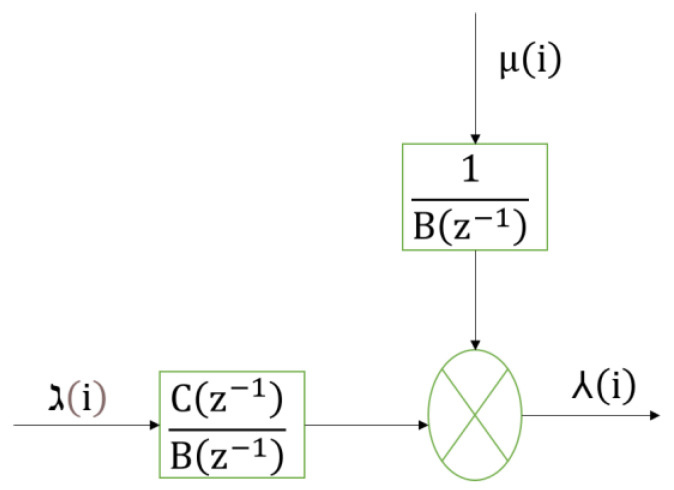
ARX model block diagram.

The ARX structure effectively model various engineering and applied sciences problems such as time series prediction, pneumatic positioning system, wheeled robots, MIMO systems, and behavior modeling [78,79,80,81,82]. The block diagram of the ARX model is presented in Figure 2, where $B\left(z^{-1}\right)$ and $C\left(z^{-1}\right)$ are polynomials with a degree $n_{b}$ and $n_{c}$ respectively, and given in (1) and (2). $\mu \left(i\right)$ is random noise, $ℷ\left(i\right)$ is the input, and $⅄\left(i\right)$ is the output

(1) $$\;B\left(z^{-1}\right)=1+b_{1}z^{-1}+b_{2}z^{-2}+\cdots +b_{n_{b}}z^{-n_{b}}$$

(2) $$\;C\left(z^{-1}\right)=c_{1}z^{-1}+c_{2}z^{-2}+\cdots +c_{n_{c}}z^{-n_{c}}$$

The output from Figure 2 is presented in (3).

(3) $$⅄\left(i\right)=\frac{C\left(z^{-1}\right)}{B\left(z^{-1}\right)}ℷ\left(i\right)+\frac{1}{B\left(z^{-1}\right)}\mu \left(i\right)$$

Solving (3) as presented in (4)

(4) $$\;B\left(z^{-1}\right)⅄\left(i\right)=C\left(z^{-1}\right)ℷ\left(i\right)+\mu \left(i\right)$$

(4) can be rearranged as presented in (5)

(5) $$⅄\left(i\right)=\left[1-B\left(z^{-1}\right)\right]⅄\left(i\right)+C\left(z^{-1}\right)ℷ\left(i\right)+\mu \left(i\right)$$

The information vectors are defined in (6) and (7).

(6) $$\;{ⱷ}_{b}\left(i\right)=\left[-⅄\left(i-1\right),-⅄\left(i-2\right),\cdots ,-⅄\left(i-n_{b}\right)\right]$$

(7) $$\;{ⱷ}_{c}\left(i\right)=\left[-ℷ\left(i-1\right),-ℷ\left(i-2\right),\cdots ,-ℷ\left(i-n_{c}\right)\right]$$

The parameter vectors are presented in (8) and (9).

(8) $$\;b=\left[b_{1},b_{2},\cdots ,b_{n_{b}}\right]$$

(9) $$\;c=\left[c_{1},c_{2},\cdots ,c_{n_{c}}\right]\;$$

The overall information and parameter vectors are given in (10) and (11), respectively.

(10) $$\;ⱷ\left(i\right)=\left[\begin{array}{cc} {ⱷ}_{b}\left(i\right) & {ⱷ}_{c}\left(i\right) \end{array}\right]$$

(11) $$\;£=\left[\begin{array}{cc} b & c \end{array}\right]\;$$

The identification model of ARX system presented in Figure 1 is given in (12), and the parameter vector of ARX provided in (11) is estimated through the proposed optimization heuristics.

(12) $$\;⅄\left(i\right)={ⱷ}^{T}\left(i\right)£+\mu \left(i\right)$$

## 3. An Improved Chaotic Grey Wolf Optimization (ICGWO)

GWO is a recently proposed metaheuristic inspired by social hierarchy and the hunting behavior of grey wolves. Grey wolves are apex predators and prefer to live in a pack size of five to twelve on average with a strict dominant hierarchy. The leaders are male and female and responsible for decisions regarding hunting, the place for sleep, time for waking up, etc. The leader wolf is dominant, and the pack should follow his/her orders. The leader wolves may not be the strongest, but it is the best in terms of management. Hunting is the second interesting behavior of grey wolves after social hierarchy. The main steps of hunting in grey wolves are approaching the prey after tracking and chasing it, harassing the prey until it stops moving after pursuing and encircling, and finally, attack towards the prey.

ICGWO is an improved version of GWO for solving optimization problems. Its mathematical model is presented below.

### 3.1. Social Hierarchy

In this step, the fittest solution $\alpha_{1}$ along with the second and third fittest solutions $\alpha_{2}$ and $\alpha_{3}$, respectively, were considered. The rest of the solutions were presumed to be ω.

### 3.2. Encircling Prey

In this step, the wolves encircle the prey as presented in (13) and (14).

(13) $$\;\vec{F}=\left|\vec{E}.{\vec{X}}_{\mathrm{pr}}\left(g_{n}\right)-\vec{X}\left(g_{n}\right)\right|$$

(14) $$\;\vec{X}\left(g_{n}+1\right)={\vec{X}}_{\mathrm{pr}}\left(g_{n}\right)-\vec{Y}.\vec{F}$$

where ${\vec{X}}_{\mathrm{pr}}\left(g_{n}\right)$ is the prey’s position, and $\vec{Y}\;\mathrm{and}\;\vec{F}$ are vectors of coefficients as defined in (15) and (16).

(15) $$\vec{Y}=2\vec{y}.{\vec{s}}_{1}-\vec{y}\left|\vec{E}.{\vec{X}}_{\mathrm{pr}}\left(g_{n}\right)-\vec{X}\left(g_{n}\right)\right|$$

(16) $$\vec{E}=2.{\vec{s}}_{2}$$

where $s_{1}$ and $s_{2}$ are random vectors, and $\vec{y}$ is an improved convergence factor whose value decreases non-linearly from 2 to 0, as presented in (17).

(17) $$\vec{Y}=2-2\times \left(\frac{1}{e-1}\times \left(e^{\frac{g_{n}}{{\mathrm{Maxg}}_{n}}}-1\right)\right)$$

where ${\mathrm{Maxg}}_{n}$ is the maximum number of generations, and $g_{n}$ is the current generation.

### 3.3. Hunting

In this step, the positions from the three best solutions $\alpha_{1}$, $\alpha_{2}$, and $\alpha_{3}$ are considered, while the rest of the solutions ω were required to follow the best solutions. It is presented in (18)–(24).

(18) $${\vec{F}}_{\alpha_{1}}=\left|{\vec{E}}_{1}.{\vec{X}}_{\alpha_{1}}\left(g_{n}\right)-\vec{X}\left(g_{n}\right)\right|$$

(19) $${\vec{F}}_{\alpha_{2}}=\left|{\vec{E}}_{2}.{\vec{X}}_{\alpha_{2}}\left(g_{n}\right)-\vec{X}\left(g_{n}\right)\right|$$

(20) $${\vec{F}}_{\alpha_{3}}=\left|{\vec{E}}_{3}.{\vec{X}}_{\alpha_{3}}\left(g_{n}\right)-\vec{X}\left(g_{n}\right)\right|$$

(21) $$\;{\vec{X}}_{k1}\left(g_{n}\right)={\vec{X}}_{\alpha_{1}}\left(g_{n}\right)-{\vec{Y}}_{k1}.{\vec{F}}_{\alpha_{1}}\left(g_{n}\right)$$

(22) $$\;{\vec{X}}_{k2}\left(g_{n}\right)={\vec{X}}_{\alpha_{2}}\left(g_{n}\right)-{\vec{Y}}_{k2}.{\vec{F}}_{\alpha_{2}}\left(g_{n}\right)$$

(23) $$\;{\vec{X}}_{k3}\left(g_{n}\right)={\vec{X}}_{\alpha_{3}}\left(g_{n}\right)-{\vec{Y}}_{k3}.{\vec{F}}_{\alpha_{3}}\left(g_{n}\right)$$

(24) $$\;\vec{X}\left(g_{n}+1\right)=\frac{{\vec{X}}_{k1}\left(g_{n}\right)+{\vec{X}}_{k2}\left(g_{n}\right)+{\vec{X}}_{k3}\left(g_{n}\right)}{3}$$

where ${\vec{Y}}_{k1},{\vec{Y}}_{k2},{\;\mathrm{and}\;\vec{Y}}_{k3}\;\mathrm{were}\;\mathrm{calculated}\;\mathrm{from}\;\left(15\right),{\;\mathrm{and}\;\vec{E}}_{1}$,${\vec{E}}_{2}$, and ${\vec{E}}_{3}$ were calculated from (16).

### 3.4. Attacking

In this step, the hunting step is terminated based on $\vec{y}$ presented in (17) as it decreases non-linearly over generations for better exploration and exploitation in ICGWO.

### 3.5. Chaotic Map

**Figure 3:**
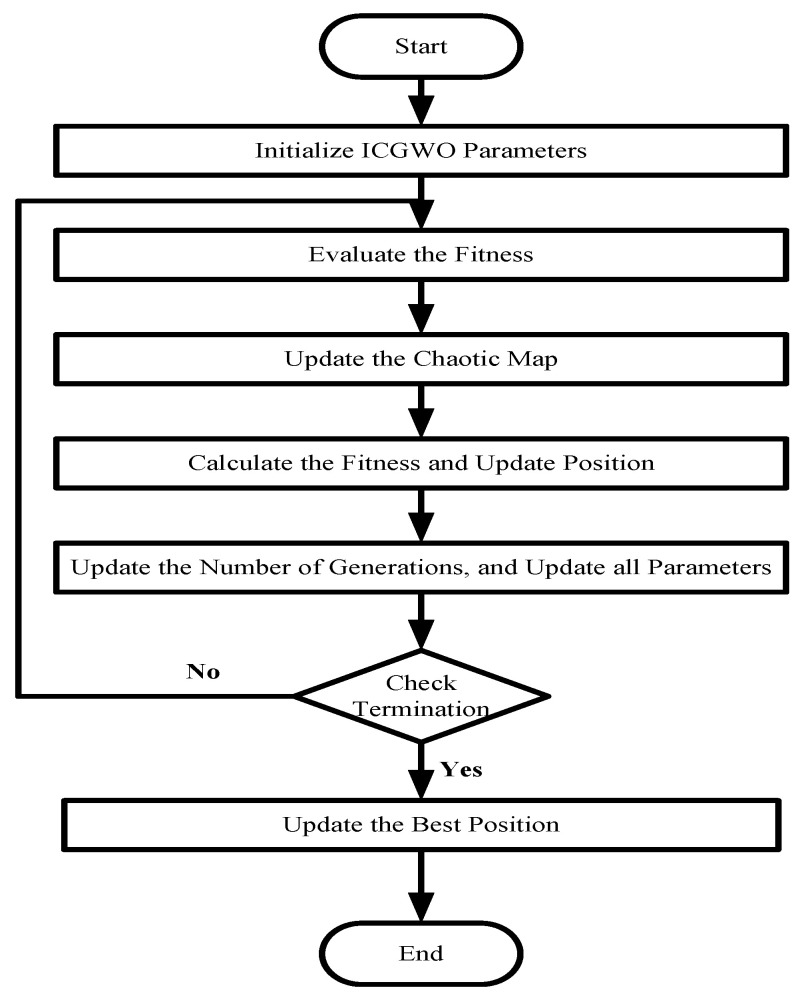
ICGWO Flowchart.

To maintain the diversity, a logistic map is used such that the algorithm avoids the local minimum values during optimization. Its mapping is presented in (25).

(25) $$\;x_{j+1}={\beta x}_{j}\left(1-x_{j}\right)$$

where β=4 for chaotic state population. The flowchart of ICGWO is shown in Figure 3.

First, the parameters of ICGWO were set. Then, the best fitness solutions were assigned to $\alpha_{1}$, $\alpha_{2}$, and $\alpha_{3}$. Afterward, ω and the logistic chaotic map were updated. Finally, parameters were updated, and an optimal solution can be obtained.

## 4. Experimental Analysis

In this section, the experimental analysis of ICGWO for the ARX model is presented. The analysis was conducted on several variations of populations ($p_{n}$), generations ($g_{n}$), and noise levels. The simulations were conducted in a MATLAB environment with zero mean unit variance input signal, and the noise signal has a normal distribution with constant variance. The accuracy is evaluated in terms of fitness given in (26).

(26) $$\mathrm{FF}=\mathrm{mean}\;{\left(⅄-\hat{⅄}\right)}^{2}$$

where $\hat{⅄}$ is the estimated/approximated response and ⅄ is the true/actual response. The model used for simulations is taken from [90] and presented in (27)–(28).

(27) $$B\left(z^{-1}\right)=1-1.53z^{-1}+0.66z^{-2}$$

(28) $$C\left(z^{-1}\right)=0.25z^{-1}+0.30z^{-2}$$

**Figure 4:**
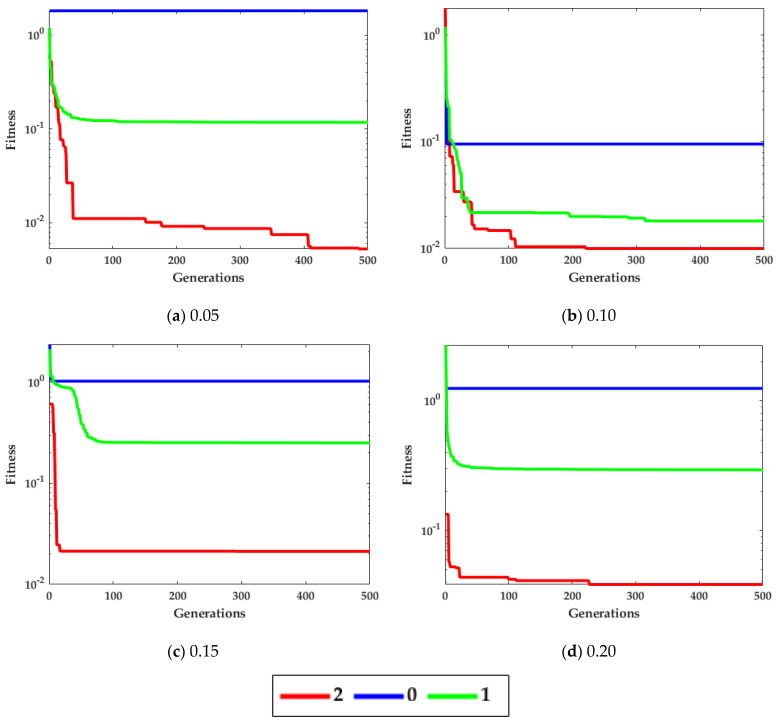
ICGWO curves with respect to convergence factor.

The noise $\mu \left(i\right)$ is taken as white Gaussian with variances [0.05, 0.10, 0.15, 0.20]. The performance is evaluated on the population $(p_{n}$ = 10, 30) and generations ($g_{n}$ = 200, 500). Figure 4 shows the curves for different variations of the convergence factor of ICGWO. It is perceived from Figure 4a–d that upon ICGWO balances between exploration and exploitation when $\vec{y}$ decreases nonlinearly from 2 to 0 for all noise variations. Table 1 shows the difference between variants of GWO. In AGWO, adaptive convergence is incorporated in GWO. This convergence factor decreases nonlinearly from 2 to 0. In CGWO, a logistic chaotic map is incorporated in GWO for balance between exploration and exploitation. In ICGWO, both the adaptive convergence factor and chaotic map were incorporated in GWO.

**Figure 5:**
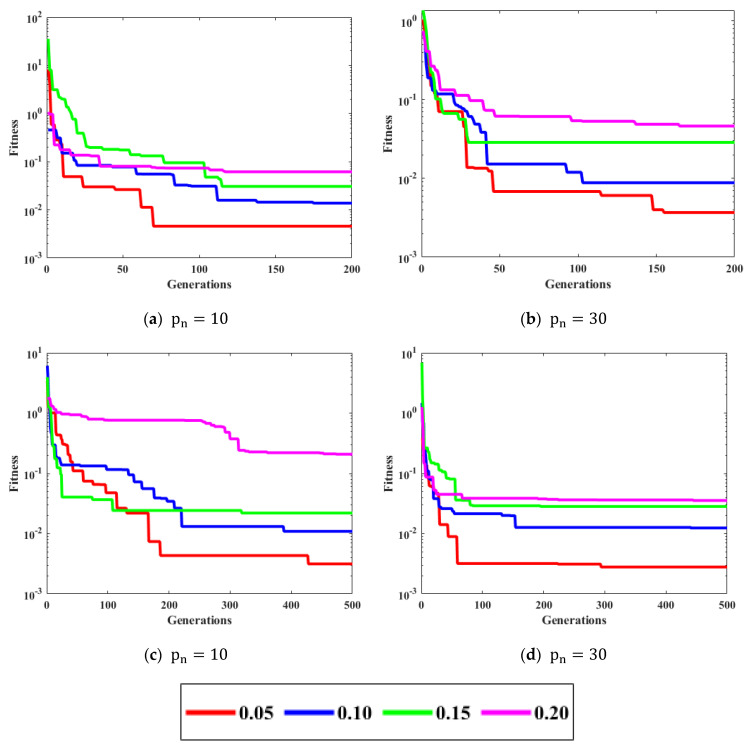
ICGWO convergence curves with respect to noise variances.

Figure 5 displays the convergence curves of ICGWO for all noise variances. It is perceived from Figure 5a–d that upon increasing $p_{n}$ and $g_{n}$, the value of fitness reduces. However, for high noise variances, the fitness also increases.

Table 2, Table 3, Table 4 and Table 5 exhibit the performance of ICGWO with GWO, AGWO, and CGWO for best-estimated parameters and corresponding fitness values for [0.05, 0.10, 0.15, 0.20] noise levels. It is notable that for low noise level, i.e., 0.05, the outcomes of ICGWO are better in contrast to higher noise levels. It is also notable from Table 2, Table 3, Table 4 and Table 5 that the best fitness for 0.05, 0.10, 0.15, and 0.20 noise levels are 0.00222, 0.00863, 0.01946, and 0.03440, respectively. Therefore, it is established from Table 2, Table 3, Table 4 and Table 5 that the fitness of ICGWO reduces with an increase in noise levels.

**Figure 6:**
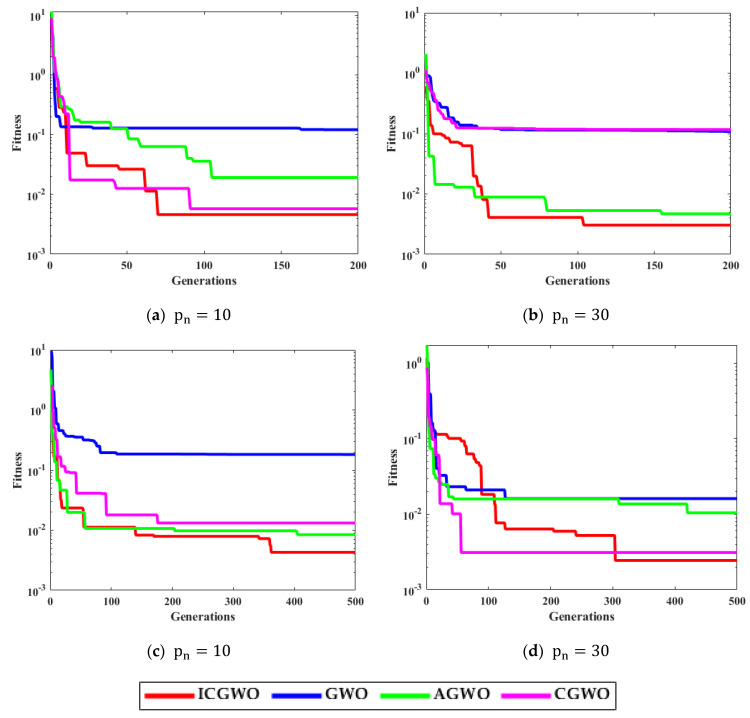
Convergence curves with respect to 0.05 noise.

**Figure 7:**
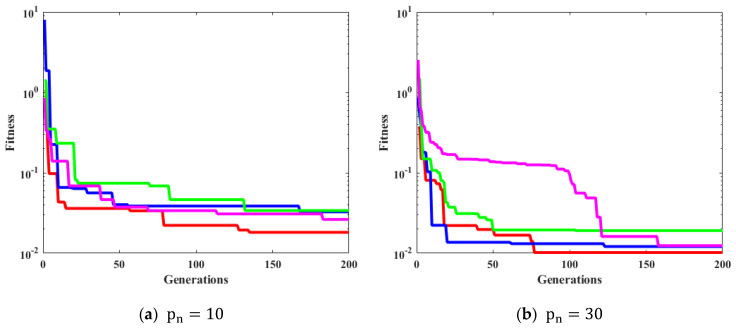

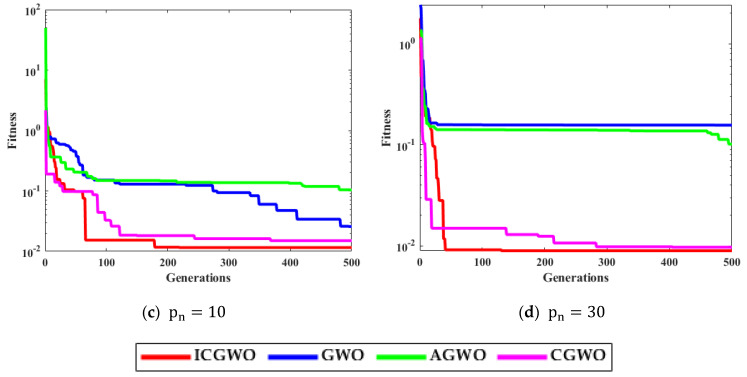
Convergence curves with respect to 0.10 noise.

**Figure 8:**
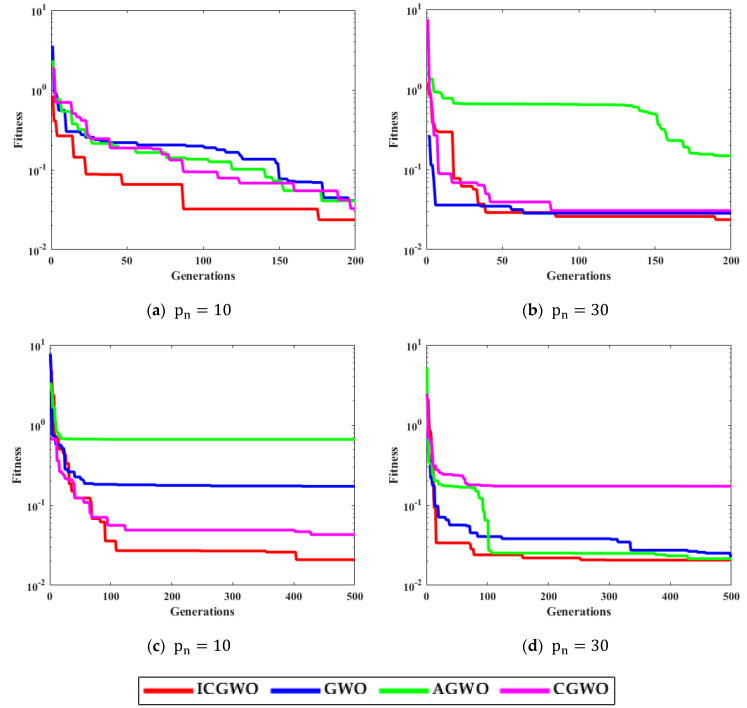
Convergence curves with respect to 0.15 noise.

**Figure 9:**
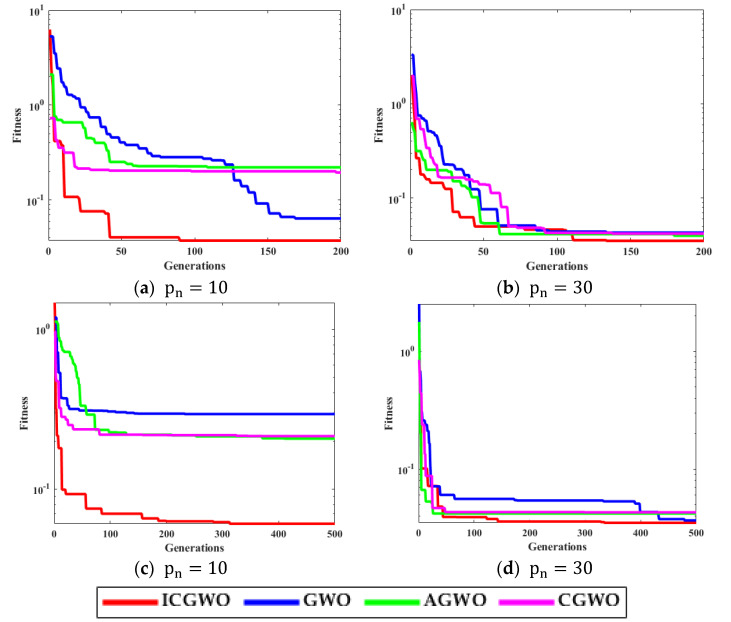
Convergence curves with respect to 0.20 noise.

Figure 6, Figure 7, Figure 8 and Figure 9 confirm the convergence of ICGWO with GWO, AGWO, and CGWO for all levels of noise. Figure 6 shows the convergence for the 0.05 noise level. Figure 7 shows the convergence for the 0.10 noise level. Similarly Figure 8 and Figure 9 shows the convergence for 0.15 and 0.20 noise levels respectively. It is notable from Figure 6, Figure 7, Figure 8 and Figure 9 that upon the rise in noise levels, the fitness value increases. For the noise levels shown in Figure 6, Figure 7, Figure 8 and Figure 9, it is evident that the convergence of ICGWO is consistent and it accomplishes the lowest fitness value than GWO, AGWO, and CGWO for all scenarios.

**Figure 10:**
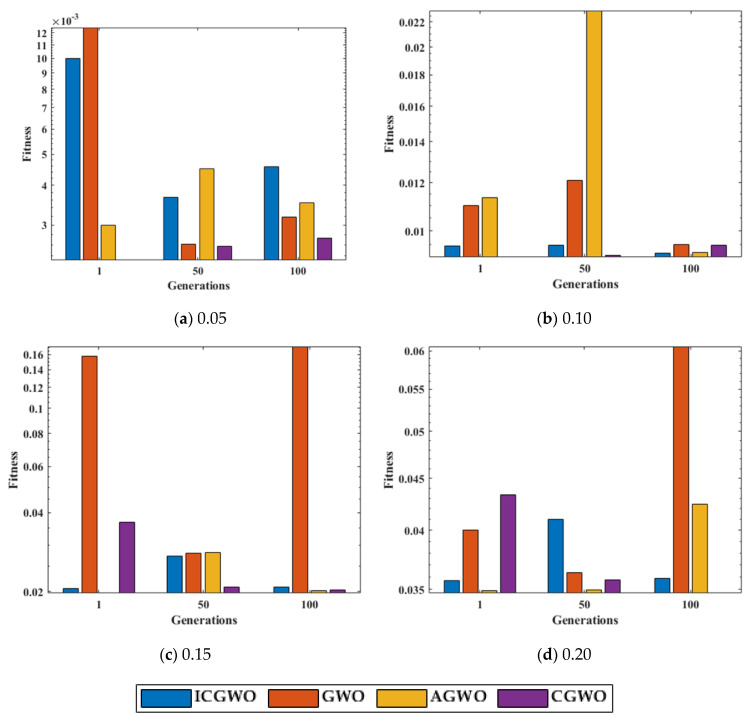
Run# fitness value comparison of ICGWO with GWO, AGWO, and CGWO at $p_{n}=30$ and $g_{n}=500$.

A statistical study of ICGWO against GWO, AGWO, and CGWO at $p_{n}=30$, $g_{n}=500$ for 100 independent runs is displayed in Figure 10. Figure 10a shows the performance for 0.05 noise level. Similarly, Figure 10b–d shows the performance for noise levels 0.10, 0.15 and 0.20 respectively. It is perceived from Figure 10 that the fitness value of ICGWO against GWO, AGWO, and CGWO is lower on run#1, run#50, and run#100 for all levels of noise.

**Figure 11:**
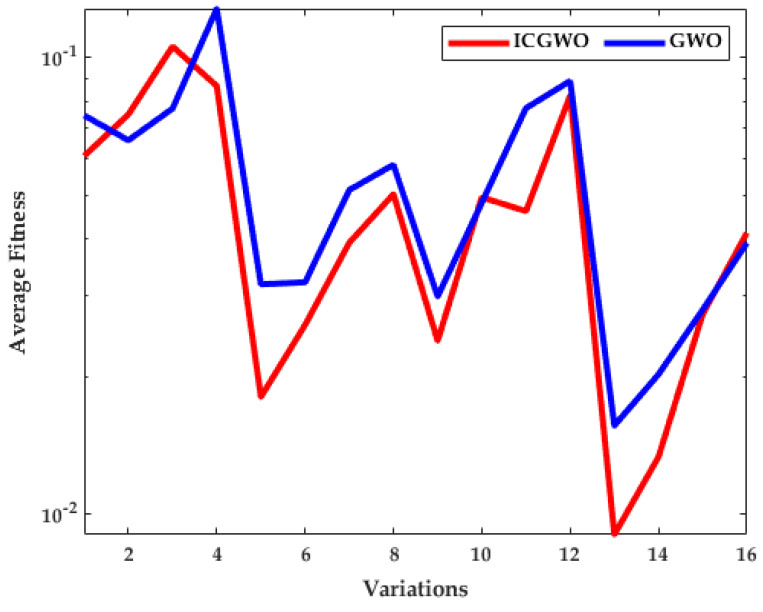
ICGWO vs. GWO statistical curve with respect to average fitness.

**Figure 12:**
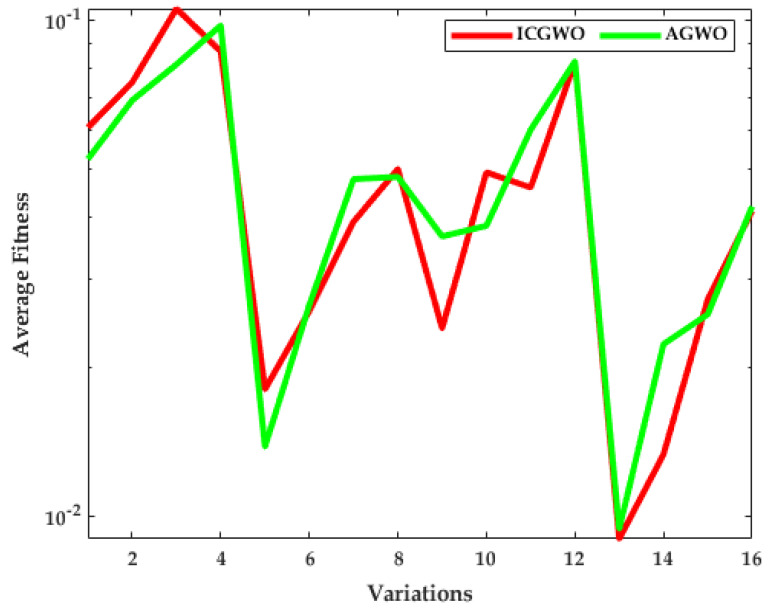
ICGWO vs. AGWO statistical curve with respect to average fitness.

**Figure 13:**
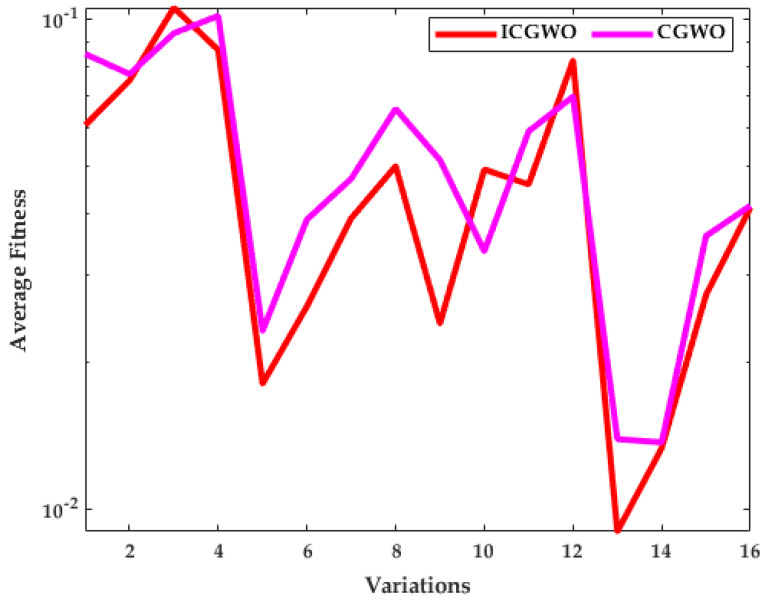
ICGWO vs. CGWO statistical curve with respect to average fitness.

The investigation of ICGWO is further explored in terms of average fitness values for all scenarios of $p_{n}$ and ${\;g}_{n}$, as revealed in Figure 11, Figure 12 and Figure 13. Figure 11 shows the values of average fitness for noise variances = 0.05, 0.10, 0.15, 0.20], population $(p_{n}$ = 10, 30) and generations ($g_{n}$ = 200, 500) between ICGWO and GWO. Similarly, Figure 12 and Figure 13 show these variations between ICGWO vs AGWO and ICGWO vs CGWO respectively. In Figure 11 it is established that ICGWO achieves the lowest fitness values than GWO for all sixteen variations. In Figure 12, the performance of ICGWO is still more significant than AGWO. Similarly, ICGWO outperforms CGWO in all variations in Figure 13. Therefore, it is established from Figure 11, Figure 12 and Figure 13 that ICGWO accomplishes a better performance than GWO, AGWO, and CGWO for all scenarios.

### Application to LD-Didactic Temperature Process Plant

To validate the performance of the proposed methodology, an ARX-based LD-Didactic temperature process plant model is considered. The authors of [91] described that the LD-Didactic temperature process consists of the pre-processing unit, a model selection unit, model estimation, and model validation. During pre-processing, noise data is filtered from temperature data. In model selection, ARX is considered due to low complexity [91]. The true parameters of ARX structure reflecting the actual dynamics of temperature process system are taken from the real time experimentation [91]. These parameters are presented in (29). The model is estimated by using variants of GWO i.e., AGWO, CGWO, and ICGWO.

(29) $$\tau ={\left[-0.2532,-0.7594,\;98.7000,-97.6400\right]}^{T}$$

**Figure 14:**
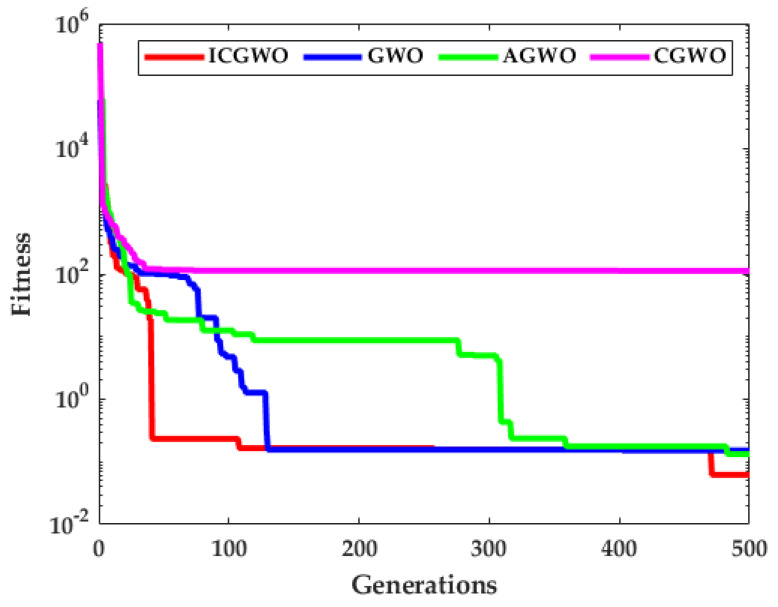
Convergence curves for LD-Didactic temperature process plant model.

The convergence curves for all noise levels at $p_{n}=30$, noise level = 0.05, and $g_{n}=500$ is displayed in Figure 14.

The results presented in Figure 14 further validate the inferences drawn form the detailed analyses of the numerical example that the proposed ICGWO provides better a performance in comparison with the conventional GWO, AGWO, and simple CGWO counterparts for parameter estimation of the temperature process plant model.

## 5. Conclusions

In this article, the strength of GWO and its various variants CGWO, AGWO, and ICGWO is exploited for parameter estimation of the ARX structure required to model various engineering and applied sciences processes. The decision parameters of the ARX model were optimized over various populations, generations, and noise levels. The logistic chaotic map along with the improved convergence factor were fused in GWO. The ICGWO is robust, accurate, and convergent for the parameter estimation of the ARX system. The convergence plots and statistical analysis through the ample number of autonomous trials confirmed that ICGWO performs better in terms of convergence and robustness as compared to conventional counterparts of the standard GWO, an improved GWO, and a simple chaotic GWO. The accurate estimation of ARX parameters reflecting the LD-Didactic temperature process plant model further validates the better performance of ICGWO. Future studies can extend the application of the proposed scheme to solve problems such as PV solar panels, constraint-preserving mixers, and real-time estimation of harmonics in nonlinear loads [92,93,94,95,96].

## Figures and Tables

**Table 1:** Parameters setting.

Method	Parameter
GWO	$\vec{y}=2$, decreases linearly to 0
AGWO	$\vec{y}=2$, decreases non-linearly to 0
CGWO	$\vec{y}=2$, decreases linearly to 0 with the chaotic map given in (25)
ICGWO	$\vec{y}=2$, decreases non-linearly to 0 with the chaotic map given in (25)

**Table 2:** Parameters estimates with respect to $g_{n}$ and $p_{n}\;$ at 0.05 noise level.

Methods	$g_{n}$	$p_{n}$	Parameters				Best Fitness
			$b_{1}$	$b_{2}$	$c_{1}$	$c_{2}$	
GWO	200	10	−1.5201	0.6553	0.2973	0.2588	0.00409
		30	−1.5418	0.6695	0.2617	0.2880	0.00232
	500	10	−1.5435	0.6772	0.2232	0.3523	0.00235
		30	−1.5542	0.6810	0.2287	0.3179	0.00219
AGWO	200	10	−1.4913	0.6427	0.2357	0.3969	0.00428
		30	−1.5459	0.6733	0.2059	0.3416	0.00229
	500	10	−1.5658	0.6916	0.2346	0.3067	0.00228
		30	−1.5339	0.6619	0.2320	0.3209	0.00218
CGWO	200	10	−1.5785	0.7069	0.2165	0.3121	0.00293
		30	−1.5304	0.6617	0.2482	0.3239	0.00223
	500	10	−1.5496	0.6705	0.2117	0.2990	0.00285
		30	−1.5517	0.6775	0.2176	0.3160	0.00231
ICGWO	200	10	−1.5363	0.6676	0.3003	0.2620	0.00294
		30	−1.5345	0.6664	0.2631	0.3037	0.00226
	500	10	−1.5363	0.6675	0.2230	0.3278	0.00241
		30	−1.5495	0.6806	0.2308	0.3242	0.00222
True Parameters			−1.5300	0.6600	0.2500	0.3000	0

**Table 3:** Parameters estimates with respect to $g_{n}$ and $p_{n}\;$ at 0.10 noise level.

Methods	$g_{n}$	$p_{n}$	Parameters				Best Fitness
			$b_{1}$	$b_{2}$	$c_{1}$	$c_{2}$	
GWO	200	10	−1.5547	0.6779	0.2139	0.3290	0.00886
		30	−1.559	0.6862	0.2218	0.3388	0.00879
	500	10	−1.5729	0.6983	0.2152	0.3265	0.00884
		30	−1.5298	0.6582	0.2119	0.3581	0.00875
AGWO	200	10	−1.5088	0.6453	0.2003	0.3967	0.00899
		30	−1.5602	0.6902	0.2292	0.3390	0.00867
	500	10	−1.5698	0.6989	0.2378	0.3221	0.00881
		30	−1.5425	0.6681	0.2086	0.3481	0.00878
CGWO	200	10	−1.5740	0.7116	0.2677	0.3247	0.00975
		30	−1.5661	0.6938	0.2319	0.3349	0.00892
	500	10	−1.5030	0.6384	0.2214	0.3758	0.00892
		30	−1.5507	0.6806	0.2144	0.3549	0.00861
ICGWO	200	10	−1.5907	0.7213	0.2108	0.3339	0.00975
		30	−1.5511	0.6785	0.2473	0.3088	0.00881
	500	10	−1.5486	0.6831	0.2512	0.3257	0.00889
		30	−1.5317	0.6641	0.2405	0.3409	0.00863
True Parameters			−1.5300	0.6600	0.2500	0.3000	0

**Table 4:** Parameters estimates with respect to $g_{n}$ and $p_{n}\;$ at 0.15 noise level.

Methods	$g_{n}$	$p_{n}$	Parameters				Best Fitness
			$b_{1}$	$b_{2}$	$c_{1}$	$c_{2}$	
GWO	200	10	−1.5511	0.6743	0.2067	0.3709	0.02077
		30	−1.5597	0.6875	0.2056	0.3773	0.01984
	500	10	−1.5266	0.6530	0.2330	0.3289	0.02018
		30	−1.5427	0.6776	0.2099	0.3823	0.01940
AGWO	200	10	−1.5655	0.6914	0.1608	0.3953	0.01997
		30	−1.5164	0.6568	0.2318	0.3900	0.01959
	500	10	−1.5524	0.6787	0.2076	0.3679	0.01975
		30	−1.5420	0.6715	0.2111	0.3621	0.01943
CGWO	200	10	−1.5034	0.6401	0.2316	0.3693	0.01994
		30	−1.5457	0.6790	0.2354	0.3592	0.01944
	500	10	−1.5394	0.6723	0.1991	0.3866	0.01938
		30	−1.5129	0.6483	0.2231	0.3865	0.01951
ICGWO	200	10	−1.5041	0.6408	0.1903	0.4027	0.02031
		30	−1.5163	0.6495	0.2248	0.3755	0.01953
	500	10	−1.5496	0.6803	0.2131	0.3565	0.01958
		30	−1.5382	0.6739	0.1925	0.4079	0.01946
True Parameters			−1.5300	0.6600	0.2500	0.3000	0

**Table 5:** Parameters estimates with respect to $g_{n}$ and $p_{n}\;$ at 0.20 noise level.

Methods	$g_{n}$	$p_{n}$	Parameters				Best Fitness
			$b_{1}$	$b_{2}$	$c_{1}$	$c_{2}$	
GWO	200	10	−1.5064	0.6478	0.2525	0.3872	0.03496
		30	−1.5504	0.6782	0.1934	0.3844	0.03470
	500	10	−1.5498	0.6788	0.1838	0.3972	0.03464
		30	−1.5342	0.6703	0.2040	0.4188	0.03446
AGWO	200	10	−1.5016	0.6498	0.2500	0.4098	0.03550
		30	−1.5572	0.6877	0.2002	0.3906	0.03461
	500	10	−1.5637	0.6967	0.2228	0.3752	0.03483
		30	−1.5370	0.6689	0.1895	0.4069	0.03444
CGWO	200	10	−1.4745	0.6126	0.1896	0.4320	0.03577
		30	−1.5228	0.6583	0.1983	0.4179	0.03437
	500	10	−1.5052	0.6419	0.2009	0.4272	0.03460
		30	−1.5305	0.6617	0.1899	0.4073	0.03449
ICGWO	200	10	−1.5753	0.7045	0.1642	0.4106	0.03520
		30	−1.5329	0.6646	0.1877	0.4102	0.03445
	500	10	−1.5610	0.6916	0.1865	0.4064	0.03470
		30	−1.5261	0.6627	0.2060	0.4179	0.03440
True Parameters			−1.5300	0.6600	0.2500	0.3000	0

## Data Availability

Not applicable.
